# Neonatal hemolytic disease due to anti-Diego^a^ antibody: a case report

**DOI:** 10.1186/s13256-022-03516-2

**Published:** 2022-07-13

**Authors:** Yangxi Fu, Ying Liu, Zhenzhen Yang, Yinghua An, Jun Su, Shuli Hu, Lingying Luo

**Affiliations:** 1 Department of Respiratory Medicine, Baoji Maternity and Child Health Care Hospital, Children’s Hospital of Baoji, Affiliated Hospital of Shaanxi University of Chinese Medicine, Baoji, 721000 Shaanxi China; 2 Department of Blood Transfusion, Baoji Maternity and Child Health Care Hospital, Children’s Hospital of Baoji, Affiliated Hospital of Shaanxi University of Chinese Medicine, Baoji, 721000 Shaanxi China; 3 Department of Neonatology, Zhuhai Hospital of Integrated Traditional Chinese and Western Medicine, The Second People’s Hospital of Zhuhai, Zhuhai, 519020 Guangdong China; 4 Department of Neonatology, Baoji Maternity and Child Health Care Hospital, Children’s Hospital of Baoji, Affiliated Hospital of Shaanxi University of Chinese Medicine, Baoji, 721000 Shaanxi China

**Keywords:** Anti-Diego^a^ antibody, Neonatal hemolytic disease, Case report

## Abstract

**Background:**

The Diego^a^ antigen commonly occurs in certain Asian and South American Indian populations. In general, hemolysis caused by anti-Diego^a^ antigen is not severe, and exchange transfusion is rarely needed. Here, we report a neonate with moderate hemolytic disease caused by anti-Diego^a^ antigen in the Baoji area of China.

**Case presentation:**

A 39-week gestation male newborn of Han nationality was delivered by second cesarean section because of scarred uterus. The newborn’s birth weight was 3700 g with an Apgar score of 9. Four hours after delivery, transcutaneous bilirubin test revealed a level of 17 mg/dl. After 23 hours, the neonate developed anemia and hyperbilirubinemia. Bacterium, virus and other pathogens, as well as tests for arcuate and glucose-6-phosphate dehydrogenase, were all negative. Direct antiglobulin test of the neonate was positive. Diego^a^ antigens of the baby and his father were positive, while his mother was negative. The newborn was successfully cured with phototherapy and one-dose intravenous injection of human albumin.

**Conclusions:**

It is important to consider and test for the anti-Diego^a^ antibody in cases of hemolytic disease of the newborn of the Han ethnicities of China.

## Introduction

The Diego blood group system was first introduced in 1955 by a case of hemolytic disease of the newborn caused by anti-Diego^a^ antibodies (anti-Di^a^) [1]. The Diego blood group system is composed of mainly three sets of antithetical antigens: Di^a^/Di^b^, Wr^a^/Wr^b^, and Wu/DISK [1]. Subsequent studies have found that the Diego blood group antibodies (for example, anti-Di^a^ and anti-Di^b^) can cause hemolytic transfusion reaction (HTR) and hemolytic disease of the fetus and newborns (HDFN or HDN) [2].

The distribution frequency of Di^a^ antigen in different ethnic groups and regions is known to be very different. Genetic study revealed that Di^a^ antigen was relatively common among Asians of Mongoloid origin and South American Indians, as compared with Caucasians and Blacks [3]. Only a few publications reported that Di^a^ was a low-frequency antigen in Europe, such as 0.89% in Berlin [4] and 0.46% in Poland [5]. One study in a South Texas community demonstrated a relatively high frequency (2.6–4%) in previously transfused patients from an area with 20–54% Mexican donors [6]. Another study showed that Di^a^ incompatibility among the southern Thais (0.93%) was lower than among the central Thais (3.49%) [7].

Anti-Di^a^ antigen has been reported as being responsible for moderate to severe HDN [5, 8, 9]; however, it rarely caused a fatal hemolytic transfusion reaction [10]. In this paper, we report a case of moderate HDN caused by Di^a^ antibody. Fortunately, blood transfusion and red blood cell suspension injection were not required.

His parent gave written informed consent for publication. This manuscript adheres to the applicable EQUATOR guideline: CARE checklist.

## Case presentation

A 39-week gestation male newborn of Han nationality, delivered by second cesarean section because of scarred uterus, was born in the sixth hospital of the Baoji area. The newborn’s birth weight was 3700 g with an Apgar score of 9. His mother had no history of blood transfusion. This was her second pregnancy; her first child was a healthy 7-year-old girl. Routine prenatal examination for irregular antibodies had never been performed during her second pregnancy. The condition of the baby stabilized, and there were no findings of fetal distress. Four hours after birth, the baby was found to have obvious jaundice and transcutaneous bilirubin test reached a level of 17 mg/dl in the sixth hospital of the Baoji area. Then he was immediately sent to the neonatal intensive care unit of our hospital for further evaluation and monitoring, and the transcutaneous bilirubin level was 16.5 mg/dl on admission and first treated with intensive phototherapy for 16 hours. Laboratory results were as follows (23 hours after birth): red blood cell (RBC) 3.3 × 10^12^ cells/L, white blood cell count 15.73 × 10^9^ cells/L, hemoglobin 12.9 g/L, hematocrit 36%, platelet count 223 × 10^9^/L; liver function test showed total bilirubin 13.3 mg/dl, and unconjugated bilirubin 12.6 mg/dl. C-reactive protein (CRP) was 63.5 mg/L, procalcitonin was 6.2 ng/ml, and interleukin-6 was 299.8 pg/ml. The following tests were all negative: blood culture, urinalysis, and stool (microscopic examination), cerebrospinal fluid routine, cerebrospinal fluid culture and biochemistry, tests for blood cytomegalovirus, herpes simplex virus, and other pathogens, as well as tests for arcuate and glucose-6-phosphate dehydrogenase (G-6-PD).

The newborn and his mother were typed as blood group A, RhD^+^. The subtypes of Rh blood group were classified C, c, D, E, and e. Free test and diffusion test were negative. The neonate’s red cells reacted positively (1+) in the direct antiglobulin test (DAT) including (IgG + C3d) polyclonal antibody; however, the anti-IgG test had a positive result, while anti-C3d test was negative. The irregular antibodies of the mother by microcolumn gel technology combined with anti-human globulin (IgG + C3d) were positive (IgG 1+, C3d−). Because the mother did not receive blood transfusion and gamma globulin, we speculated that the anti-IgG might be against a rare blood group system. No antibodies against red blood group system were identified in tests using commercial antibody screening blood cell (Rh-hr, Kidd, MNSs, Duffy, Lewis, P). Anti-Di^a^ was finally identified in the neonate’s serum from tests with a commercial panel of red blood cells (spectrum cells including Rh-hr, Kidd, MNSs, Duffy, Diego, Kell, Lewis, P, DO, Yt) from the Shanghai Blood Biotechnology Company, China. To confirm the anti-Di^a^, blood samples of the newborn and his parents were sent to Shanghai Blood Center and further performed using a monoclonal anti-Di^a^ and monoclonal anti-Di^b^ standard serum, respectively. Thus, father and child were positive for Di^a^ antigen, while mother was negative for Di^a^ antigen. Di^b^ antigen was negative for all of them.

The neonate was successfully treated with intensive phototherapy for 120 hours over 10 days, and received intravenous injection of 70 ml human albumin injection. The bilirubin level dropped to 8.2 mg/dl within a few days of treatment, and therefore, no blood exchange transfusion was needed because the hemolysis was not aggravated. The infant was in a healthy state and was discharged home with the following laboratory findings: RBC 3.02 × 10^12^ cells/L; hemoglobin 111 g/L; hematocrit 34.1%.

## Discussion

In the past, ABO and Rh incompatibility generally accounted for HDN. With the determination of blood group antibody titer and the use of anti-D immunoglobulin in pregnant women, fatal and severe HDN has been dramatically reduced in recent years. Owing to prenatal and postnatal maternal serum irregular antibody screening for pregnant women, the special antibodies to rare RBC surface antigens, such as Di^a^ and Di^b^, were subsequently identified. These antibodies played an important role in rare and severe HDN.

In our case, the mother had not been screened for irregular antibodies during the pregnancy. Unfortunately, postnatally, the irregular antibodies of the mother’s serum were positive, and the neonate had clinical signs of HDN with positive DAT. In the present study, the mother was negative for Di^a^ antigen, but the baby and father were positive. Anti-Di^a^ antibody is also pregnancy-induced [5, 11] and is detectable from birth [3, 12]. We speculated anti-Di^a^ antibody formation during this pregnancy, and its level increased, crossed the placental barrier, and entered fetal circulation. Quantity of fetal RBC cells attached to anti-Di^a^ antibodies might be enough to lead to postnatal clinical signs of HDN and cause positive DAT.

In general, hemolytic disease of the newborn induced by anti-Di^a^ is not severe; however, some cases may be serious enough to require exchange transfusion [11, 12]. The baby demonstrated moderate hemolysis and hyperbilirubinemia due to anti-Di^a^, similarly to cases reported previously [8, 13, 14]. Nevertheless, our findings are apparently contradictory to several other reports. Larysse and Arends, for example, could not find a case of HDN in 40 families tested, in which fathers were Di^a+^, mothers Di^a−^, and children Di^a+^ [15]. Zeljka Hundric-Haspl *et al*. reported a neonate who had no clinical signs of HDN despite positive DAT, and the pre- and postnatal screening tests of the mother were also all negative; however, anemia was diagnosed 3 weeks later, and it was caused by anti-Diego derived during pregnancy, which led to low-level Di RBC antigen [16]. Moreover, a few examples of agglutinins of anti-Di^a^ specificity have been reported in individuals with no known RBC exposure [1], the reasons for which remain unknown.

It is well known that the Di^a^ antigen is usually associated with Mongolian populations in Asia. In Japan, however, there is no significant difference in the prevalence among Mongolians, Zhuangs, Chinese–Koreans, and Japanese as reported by Komatsu *et al*. [17]. In China, the Di^a^ antigen is usually found among the Korean and Zhuang nationalities [18]. Overall, these findings confirm that the Di^a^ antigen occurs largely among people of Mongolian origin. In China, in particular, the Mongolian people are distributed mainly over Inner Mongolia, Gansu, Xinjiang, Shaanxi, and northeast China [18]. The Baoji area is located in Shaanxi Province, which belongs to the northwest region of the mainland, and the Mongolian people are scattered here. However, the baby in this case study was of a Han nationality instead of a minority, and so were his parents. who were also local residents. This suggests that the Di^a^ antigen may be present in diverse populations.

To the best of our knowledge, the present study may be the first report of this case in Shaanxi Province, China. Although antibodies to Di^a^ antigen are not the main cause of hemolytic disease of newborns in the area, such antibodies may lead to severe hemolytic reactions. When encountering hemolytic disease of the newborn caused by low-frequency antigen-antibody, it is necessary to consider the role of anti-Di^a^. It is also recommended that the screening for Di^a^ antibodies be included in routine prenatal examinations.

## Conclusion

It is important to consider the role of an anti-Di^a^ antibody in cases of hemolytic disease of the newborn in the Han populations in China. It is also recommended that antibody screenings include anti-Di^a^ screening for all pregnant women and neonates in order to prevent early complications of hemolytic disease of the newborn and potentially harmful outcomes of transfusion therapy.

## Data Availability

Not applicable.

## Abbreviations

Di^a^: Diego^a^
G-6-PD: Glucose-6-phosphate dehydrogenase
DAT: Direct antiglobulin test
HTR: Hemolytic transfusion reaction;
HDFN or HDN: Hemolytic disease of the fetus and newborns
RBC: Red blood cell
CRP: C-reactive protein
